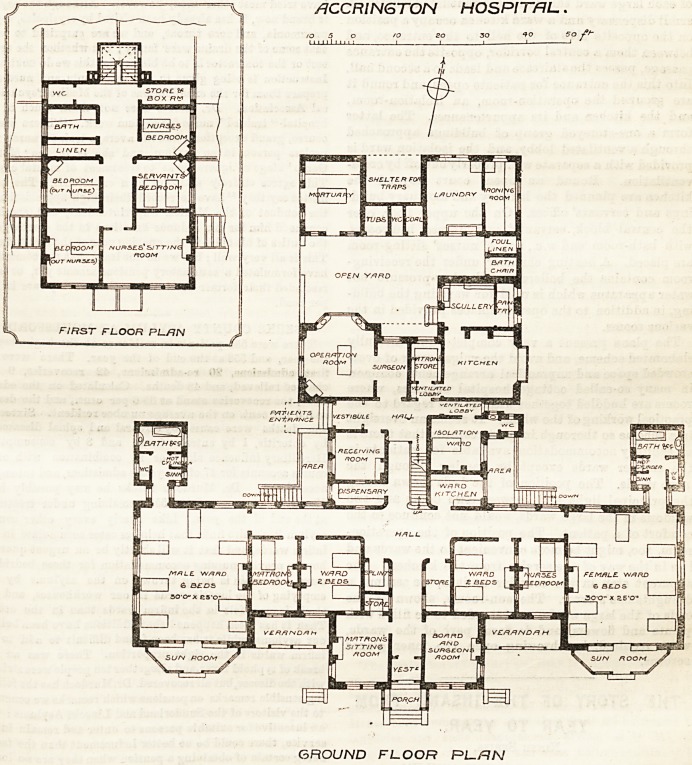# Hospital Construction

**Published:** 1896-09-05

**Authors:** 


### The Institutional Workshop.

### HOSPITAL CONSTRUCTION.

### ACCRINGTON COTTAGE HOSPITAL.

The building is pleasantly situated in an elevated position overlooking the town, and is intended to serve the requirements of the population of the im-

ACCRINGTON HOSPITAL.

FIRST FLOOR PLAN

GROUND FLOOR PLAN

mediate neighbourhood, containing factories, mills, collieries, printing, and iron works, from which the victims of accidents and other "cases" have hitherto been sent to Blackburn or Burnley. It is well set back from the road and has the advantage of an entrance from a side street in addition to that from the main thoroughfare. Of this the architects, Messrs. Hayward and Harrison, have taken advantage, and so placed the entrance and receiving-room for accidents and patients as to leave the inmates of the wards undisturbed. Ample land has been secured for the extension, if necessary, of the hospital buildings.

The main frontage of the building looks nearly south, and the principal entrance is centrally placed between the matron's sitting-room and the board-room. It is suggested in the architects' report that the latter may be used for coroners' inquests, but its position with regard to the mortuary does not seem to recommend it in this respect. Store rooms of various kinds are arranged under controll of the matron on both sides of an entrance passage leading to a hall, out of which the staircase rises to the upper part of the building, and corridors, well lighted at the sides, run right and left to the male and female wings respectively. Each of these wings comprises a large ward for six beds and a small ward for two beds, with a nurse's room between them and controlling them. A sanitary block, containing bath-room, w.c., slop-sink an d shoot for surgical dressings, and lockers for patient's clothes, open out of each large ward through cross-ventilated lobbies. A small dispensary and a ward kitchen occupy a position on the opposite side of the hall to the entrance, and between them a central corridor, opposite the entrance passage, passes the staircase and leads to a second hall. Into this the entrance for patients opens, and round it are grouped the operation-room, an isolation-room, and the kitchen and its appurtenances. The latter form a one-storeyed group of buildings approached through a ventilated lobby, and the isolation ward is provided with a separate w.c. properly cut off by cross-ventilation. Round an open court beyond the kitchen are planned the laundry and mortuary buildings and servants' offices. On the upper floor, over the central block, servants' and nurses' bed-rooms, with bath-room and w.c., and a nurses' sitting-room are placed. A heating chamber under the receiving-room contains the boilers for the low-pressure hot water apparatus which is used for warming the building, in addition to the open fireplaces provided in the various rooms.

The plans present a very complete and carefully elaborated scheme, and avoid the vulgar error of overcrowded space and unpractical arrangements common in many so-called cottage hospital schemes, where rooms are huddled together without due regard to the practical working of the whole. It seems an oversight in a scheme so thorough in most respects that there is no sanitary accommodation available for patients in the smaller wards except by passing through the arge wards. The position of the beds shown facing the principal light and between two doors and two windows in the large wards, would not conduce to the comfort of a patient. The position of the operation-room, too, might be more convenient to the wards and less in the way of the service from the kitchen. The apparent step in the corridor to it is, we assume, a draughtsman's error. The sun-rooms, shown at the ends of the large wards, are intended to be filled with plants and flowers, and to form part of the wards, when required, by throwing open the inner glazed screens.

## Figures and Tables

**Figure f1:**